# Helium Leak Rate Measurements of Flight-like Mars 2020 Sample Tubes

**DOI:** 10.1089/ast.2023.0002

**Published:** 2024-01-12

**Authors:** Jeffrey T. Osterhout, Kenneth A. Farley, Meenakshi Wadhwa, Jonathan Treffkorn, Eric Kulczycki

**Affiliations:** ^1^ NASA Jet Propulsion Laboratory, California Institute of Technology, Pasadena, California, USA.; ^2^ Division of Geological and Planetary Sciences, California Institute of Technology, Pasadena, California, USA.; ^3^ School of Earth and Space Exploration, Arizona State University, Tempe, Arizona, USA.

**Keywords:** Leak rate, Mars, Sample return, Sample tube, Seal

## Abstract

The sample tubes on board NASA's Perseverance rover are designed to contain rocks, regolith, and atmospheric gases and are hermetically sealed on the surface of Mars to minimize sample loss, alteration, and contamination. Following a robust testing program during mission development, it was determined that the helium (He) leak rates of flight-like sample tubes sealed under a range of conditions were typically no greater than ∼10^−10^ standard cubic centimeters per second (scc/s); leak rates below this value could not be measured since this is the detection limit of commercially available He leak detectors. This limit was adequate to meet mission requirements. However, some scientific objectives could be compromised by sample tube leak rates even below 10^−10^ scc/s, thus motivating a more sensitive technique for establishing leak rates. This study investigated He leak rates on six flight-like sample tubes using a static mode mass spectrometer. Room temperature He leak rates of the six sample tubes ranged from ∼8.8 × 10^−17^ to ∼4.6 × 10^−14^ scc/s. One sample tube was analyzed at eight different temperatures, ranging from -51°C to +42°C, and yielded He leak rates correlated with temperature that varied from ∼1.7 × 10^−15^ to ∼1.4 × 10^−13^ scc/s, respectively. Our results confirm and extend previous findings demonstrating that the Mars 2020 sample tube seals are likely to be very leak-tight, with leak rates <10^−13^ scc/s. These leak rates are sufficiently low that the impact of gas egress or ingress is expected to be negligible.

## 1. Introduction

The return of carefully selected and well-characterized samples from the surface of Mars represents one of the main goals of modern planetary science, and the 2023–2032 Decadal Survey *Origins, Worlds, and Life: A Decadal Strategy for Planetary Science and Astrobiology* recently highlighted Mars Sample Return (MSR) as the highest scientific priority of NASA's ongoing robotic exploration efforts (National Academies of Sciences, Engineering, and Medicine, 2022). Samples of rocks, regolith, and atmosphere are currently being studied and collected by NASA's Mars 2020 rover, Perseverance, to be cached on Mars in preparation for planned future sample retrieval missions. These returned samples would comprise rock cores, regolith, and volatiles captured in hermetically sealed sample tubes and would be utilized for a variety of scientific investigations once returned to Earth (Farley *et al.,* 2020; Meyer *et al.,* 2022).

The Perseverance rover is equipped with 43 sample tubes, 38 identical tubes for collection of rock or regolith, and five that differ only in their interior constituents for acquisition of contamination knowledge (“witness tubes”). The tubes are constructed of a titanium alloy (Ti-6Al-4V) with an external coating of white alumina to minimize solar heating, and an interior surface of titanium nitride (TiN) to minimize absorption of organic contaminants (Farley *et al.,* 2020). The empty tubes have an internal volume of ∼12.5 cm^3^, while the volume of the witness tubes is about half of that. The witness tubes contain an assembly inside a hermetically sealed compartment with a particle trap, getter foils and polished gold foils to collect contaminants (*e.g.,* Moeller *et al.,* 2021). The sample tube seals are made of a separate titanium seal cup with a gold coating, which is activated by an internal ferrule (Redmond *et al.,* 2020).

Samples of drill cores collected by the Perseverance rover are assessed for their potential return to Earth as part of the MSR campaign. On board Perseverance, the sample tubes are initially stored in the adaptive caching assembly (ACA) before being moved by the sample handling assembly (SHA) to the bit carousel. The sample tube is then placed inside a coring bit, which is used to drill and capture the rock core, along with any tailings and headspace gas, inside the tube. The SHA subsequently transfers the sample tube through multiple inspection sites before advancing it to the sealing station, where a hermetic seal is created by the placement of the ferrule into the seal cup and insertion of the ram, which exerts the necessary activation force (Redmond *et al.,* 2020). After the sample tubes are sealed, they remain on the rover to be cached together in at least one “depot” on the surface of Mars for their potential future return to Earth (Farley *et al.,* 2020; Meyer *et al.,* 2022).

To assess the preservation of samples collected and stored on Mars as well as during and after their return to Earth, it is important to characterize typical leak rates for the sample tubes. Helium (He) is generally used for detecting leaks because the method is easily performed and, due to its small atomic size, provides a worst-case estimate for the leak rate of other gases. The Mars 2020 mission requirements demand hermetic-level seals for the sample tubes with a maximum allowable He leak rate of 1.0 × 10^−8^ standard cubic centimeters per second (scc/s) at room temperature (Redmond *et al.,* 2015, 2020). Leak rate measurements below the He leak-detector detection limit of 1.0 × 10^−10^ scc/s verified that this requirement was met (Redmond *et al.,* 2020). However, even at such low leak rates, sample loss, contamination, and undesirable chemical reactions (*e.g.,* hydration/dehydration reactions, isotopic fractionation) could occur in the sample tubes (Younse *et al.,* 2014; Swindle *et al.,* 2022). Leak rates below this level are thus highly desirable and may pertain to the Mars 2020 sample tubes, but evaluation of this possibility requires a more sensitive He leak rate measurement.

The negative scientific consequences of a leaking seal include the potential loss of significant amounts of atmospheric gases during transit in space and contamination with terrestrial atmosphere after the samples are returned to Earth. There are many high-priority measurements for characterizing planetary and atmospheric evolution on Mars, including analysis of the carbon and oxygen isotope composition of CO_2_, nitrogen isotope composition of N_2_, and elemental ratios and isotopic compositions of the noble gases, among others (Swindle *et al.,* 2022). These measurements, which would be performed on samples returned from Mars, will require a robust sample tube seal to preserve the initial composition and prevent the loss (or exchange) of gases from within.

There are several variables that may affect the He leak rate of the sample tube seals, including the tube wall thickness, sealing temperature, and the contact between the seal tooth and tube wall. Additionally, leak rates could also be affected by the temperatures that the tubes experience during storage on Mars and future transport to Earth. While the sample tubes on board Perseverance were not specifically designed for capturing atmospheric gases, any rock-sourced volatiles, headspace atmosphere, or dedicated atmospheric samples may be prone to sample loss, contamination, and alteration through leaks in their seals.

Here, we report the results of measurements of He leak rates for several flight-like sample tubes and seals using a time-integration method in which He gas accumulates over hours to days via leaking from inside to outside the sample tube and is then analyzed with a static mode noble gas mass spectrometer.

The primary objectives of this study are four-fold:

(1) to establish and verify the He leak rates of flight-like sample tubes with different design metrics and sealing temperatures;
(2) to characterize leak rate variation over temperature;
(3) to better understand the factors that affect sample tube leak rates as related to Mars Sample Return; and
(4) to assess the consequences of measured He leak rates for future analyses of the gas in the tubes.

## 2. Materials and Methods

### 2.1. Mars 2020 flight-like sample tubes

**FIG. 1.**
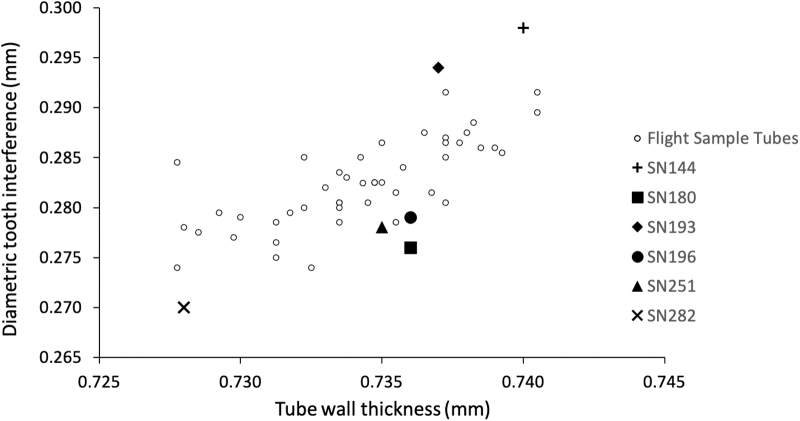
Sample tubes used in this study plotted as a function of their tube wall thickness and diametric tooth interference, in comparison to flight sample tubes carried on the Perseverance rover.

We measured six flight-like Mars 2020 sample tubes paired with their corresponding seals (Table 1; Fig. 1). These sealed sample tubes, which were provided by NASA's Jet Propulsion Laboratory (JPL), were all assembled at JPL by using aseptic flight procedures (ISO 5 cleanroom) and sealed at various temperatures under Earth ambient pressure. The sample tubes were also “nitrided” (*i.e.,* treated with a TiN coating), which provides a passivating surface that resists corrosion and minimizes organic contamination (Moeller *et al.,* 2021), and were subsequently used for drill coring as part of JPL's Packaging Qualification and Verification (PQV) testing. The sample tubes studied here were used to core a variety of rock types, including volcanic (Bishop Tuff Intermediate) as well as siliciclastic (Kramer Massive Mudstone) and chemical (China Ranch Gypsum) sedimentary rocks, and were subsequently welded to CF (ConFlat) flanges for He leak rate analyses under high vacuum. Prior to sealing, the sample tubes were baked at 150°C for 24 h, and sample tube SN144 was also thermally cycled for hermetic seal testing with temperatures ranging from roughly -130°C to +55°C (Redmond *et al.,* 2020).

**Table 1.** List of Flight-like Sample Tubes Used for He Leak Rate Measurements, Ordered According to Their Sealing Temperatures and Distinguished by Their Tube Wall Thicknesses and Diametric Tooth Interferences (Defined in Text)

Tube No.	Sealing temperature (°C)	Wall thickness (mm)	Diametric tooth interference (mm)
SN180	30	0.736	0.276
SN193	21	0.737	0.294
SN282	-30	0.728	0.270
SN144	-40	0.740	0.298
SN251	-65	0.735	0.278
SN196	-110	0.736	0.279

The sample tubes have some notable differences, as shown in Table 1, that were intended to cover the diversity of characteristics that might govern the seal leak rate. These differences include their sealing temperature (°C), tube wall thickness (measured in millimeters, mm), and diametric tooth interference (mm), which is a metric used to represent the local deformation and instantaneous expansion that occurs during seal activation and is an approximate measure of the contact between the seal tooth and sample tube wall (Redmond *et al.,* 2020). This group of tested sample tubes spans the entire range of diametric tooth interferences and tube wall thicknesses of the flight sample tubes on the Perseverance rover (Fig. 1). The actual flight sample tubes are hermetically sealed on the rover at a controlled temperature of ∼40°C.

### 2.2. Leak rate experimental design

**FIG. 2.**
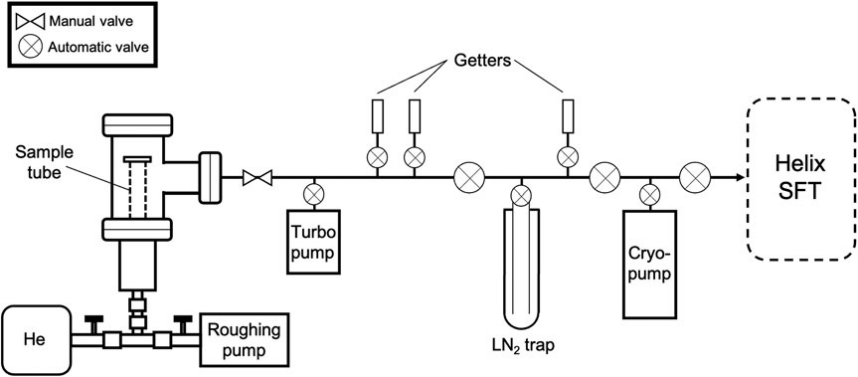
Schematic diagram of the experimental setup and gas purification system. The sample tube is first evacuated with the roughing pump before being filled with He (∼1 bar); the reservoir outside the sample tube is evacuated (∼10^−7^ torr) and subjected to static accumulation of gas across the seal.

The experimental setup for measuring the He leak rates is illustrated in Fig. 2. Helium leak rate measurements were performed at room temperature for all six of the sample tubes. For each measurement, a sample tube was connected via the CF flange to the gas purification line with vacuum held on the outside of the tube. Thus, the inside of the sample tube represents the sample-bearing side in which He gas was introduced, and the leak rate is measured as the amount of gas “leaked out” from the tube and accumulated in an initially evacuated reservoir over a specified amount of time. The chamber that houses the sample tubes (outside of seal) was first pumped down to high vacuum via a turbomolecular pump overnight before initiating any leak experiments. The inside of each sample tube was then evacuated by using a roughing pump for 1 min to ensure that the majority of the air was removed. The tube interior was filled with Ultra High Purity (UHP) He gas to atmospheric pressure (∼1 bar) via a second valve, and the process of evacuating the tube interior and filling with He was then repeated. Subsequently, we allowed static accumulation of He for a minimum of 12 h and up to 118 h into the evacuated reservoir (∼10^−7^ torr) outside the sample tube. Gases that leaked and accumulated across the sample tube seal were introduced into the purification line and processed as described below.

Following leak testing at room temperature, sample tube SN282 was measured at different temperatures, with the average temperature for each measurement ranging from roughly -51°C to +42°C. To achieve the lower temperatures required for some of these analyses, the assembly that houses the sample tube was connected to a thermocouple and set within a polystyrene container. The container was then filled with a mixture of dry ice and liquid nitrogen, varying in temperature from approximately -20°C to -80°C. Elevated temperatures were achieved by wrapping the assembly in heating tape and did not exceed ∼50°C.

### 2.3. Noble gas mass spectrometry

Gases were released by opening the manual sample isolation valve (Fig. 2) and were sequentially exposed to a heated titanium (Ti) getter, a cold SAES NP10 getter, a charcoal-bearing liquid nitrogen trap, and a final cold NP10 getter. Purified gas was then cryo-focused onto charcoal held at 12.5 K. The He was released from the charcoal at 40 K and introduced into the Thermo Helix SFT mass spectrometer for analysis. ^4^He was measured on a Faraday collector with a 1 × 10^11^ ohm resistor.

Multiple standards and blanks were run intermittently throughout each analytical session. Standards were measured to characterize and monitor instrument sensitivity. To determine the background contribution of He within the vacuum line, several blanks were analyzed in addition to the sample tube leak rate measurements. Average contribution of ^4^He during the analytical blank measurements was 0.008 nano-cubic centimeters (ncc) (±0.001, 1SD) from a total of 52 measurements made between sample runs. Thus, the detection limit for this analytical setup is approximately ∼0.01 ncc, a threshold which all (uncorrected) values for ^4^He abundance were well above (Table 2).

**Table 2.** Results of ^4^He Measurements Performed at Room Temperature for Six Flight-like Sample Tubes, Integrated Over Various Accumulation Times to Determine He Leak Rates

Tube No.	^4^He (ncc^*^)	^4^He ncc (corrected)	Accumulation time (h)	Leak rate (scc/s)
*SN144*	0.12	0.11	47.5	6.6E-16
	0.37	0.36	92.5	1.1E-15
	0.17	0.16	24.5	1.8E-15
	0.08	0.07	24.5	8.4E-16
*SN180*	0.02	0.02	24.0	2.0E-16
	0.06	0.05	72.5	2.0E-16
	0.06	0.05	72.8	2.0E-16
*SN193*	0.02	0.01	22.5	1.1E-16
	0.03	0.03	73.5	9.3E-17
	0.03	0.02	74.0	8.8E-17
*SN196*	0.03	0.03	26.5	2.7E-16
	0.05	0.05	46.0	2.7E-16
*SN251*	0.14	0.14	23.5	1.6E-15
	0.22	0.22	47.0	1.3E-15
	0.44	0.43	96.5	1.2E-15
	0.18	0.18	22.0	2.2E-15
	0.23	0.22	26.5	2.3E-15
	1.01	1.01	118.0	2.4E-15
	0.23	0.22	26.5	2.3E-15
	0.43	0.42	47.8	2.5E-15
*SN282*	3.57	3.56	22.0	4.6E-14
	15.66	15.65	96.5	4.5E-14
	3.51	3.50	21.5	4.6E-14
	4.04	4.03	25.5	4.5E-14
	7.26	7.26	45.5	4.5E-14

The measured ^4^He amounts were corrected by subtraction of blank values. Exact accumulation times (>12 h) were chosen for convenience.

^*^ nano (10^−9^) cubic centimeters

To account for He gas contribution from virtual leaks, and leaks to air associated with the experimental setup, measurements of ^4^He were also performed for sample tubes filled with UHP tank nitrogen (N_2_). Virtual leaks arise from He gas trapped in cavities within the vacuum chamber containing the sample tube, which could be interpreted as leaks (or outgassing) coming from the sample tube during analysis. The virtual leak rate was determined for each sample tube by analyzing the ^4^He concentration after filling the tube with N_2_ gas. Overall, the virtual He leak rate ranged from 0.0003 to 0.0019 ncc/h (8.3 × 10^−17^ to 5.3 × 10^−16^ scc/s) for the six sample tubes. Therefore, the lowest detectable leak rate is determined by both the average of the blank measurements and the virtual leak rate of ^4^He accumulating across the sample tube seal over time. The measured leak rates were corrected by subtracting the amount of ^4^He determined from the average of the blanks analyzed with each sample tube. However, to provide a conservative upper estimate on the seal leak rates, the contribution of ^4^He from the virtual leaks was not included in this correction.

## 3. Results

**FIG. 3.**
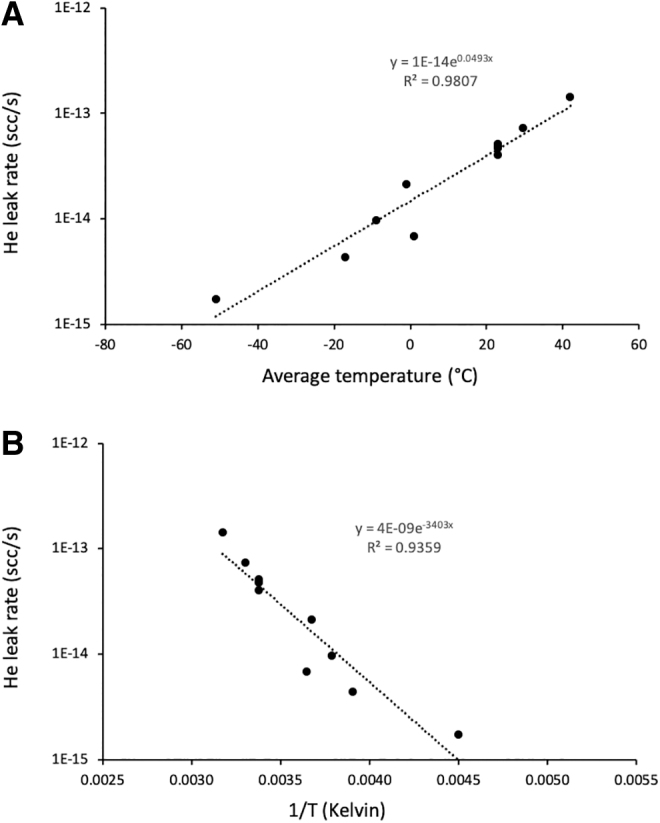
He leak rate data for sample tube SN282 over a range of temperatures. (**A**) Leak rate plotted against the average temperature that the sample tube experienced during static accumulation of He; (**B**) Leak rate plotted against inverse of the absolute temperature; linearity on this plot suggests a thermally activated process. Vertical axes are presented on a logarithmic scale.

Helium leak rate measurements for the six sample tubes analyzed at room temperature (∼23°C) are summarized in Table 2. Replicated leak rate analyses are fairly consistent for a given tube but vary between the individual tubes. All leak rates are much lower than the previously established upper limit of 1.0 × 10^−10^ scc/s. For the sample tube analyzed at different temperatures potentially seen during transit to Earth, the leak rate appears to increase with temperature. The measured He leak rates versus the average temperatures experienced by this sample tube are shown in Fig. 3.

## 4. Discussion

The sealed sample tubes will be exposed to a range of temperatures (and ambient pressures) during their storage on Mars and planned return to Earth. Changes in temperature and thermal cycling represent important environmental challenges, and early testing of the hermetic seals included analysis of He leak rates for tubes that were thermally cycled from -135°C to +70°C, and over a range of ambient pressures (from vacuum to ∼1 bar), without a measurable change in seal performance (Redmond *et al.,* 2020). Additional tests determined that the seals behave similarly for leaks into and out of the sample tube. The present study analyzed He gas leaking from inside to outside the sample tube, which is the expectation during transit from Mars to Earth. Once on Earth, however, the relative pressure gradient would lead to leakage of terrestrial atmosphere into the tubes over time.

In addition to the different sample tube characteristics noted in Table 1, the ambient temperature of the sample tube and seal assembly may also affect their leak rates. The Mars 2020 mission has determined a maximum allowable flight temperature (AFT) of 50°C for the rover and samples (Farley *et al.,* 2020), whereas MSR has a preliminary maximum temperature of 30°C for the sample tubes during their return to Earth, with certain exceptions (Tait *et al.,* 2022). There is no minimum sample temperature requirement. As can be seen in Fig. 3, there exists a strong correlation between the measured He leak rate and the average temperature during static accumulation of He. Leak rates are lower for analyses performed below room temperature (23°C) and higher for analyses above this temperature. This relationship could potentially be used to establish the leak rate consequences for heating sample tubes prior to opening, should that method be considered for sterilization. The observed differences in He leak rates can likely be attributed to one or more of the following: (1) thermal expansion or contraction of the sample tube seal with changing temperatures; (2) increasing average kinetic energy of He molecules with increasing temperature (and pressure); and (3) differing contributions of ^4^He from virtual leaks associated with increasing or decreasing temperatures.

**FIG. 4.**
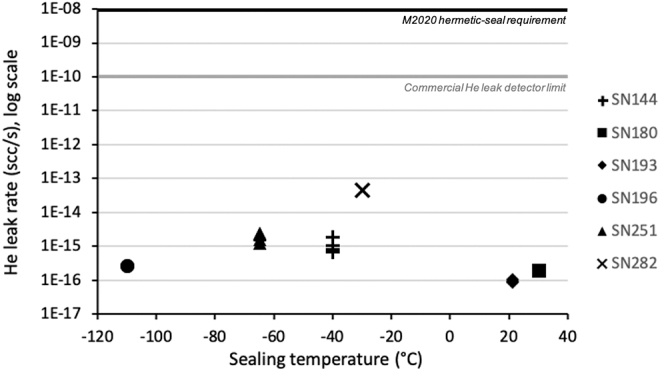
Results of measurements of He leak rates for six flight-like sample tubes with various sealing temperatures. The vertical axis is plotted on a logarithmic scale; some data points overlap such that they are indistinguishable on this graph.

Overall, the flight-like sample tubes studied here are characterized by exceptionally low He leak rates, typically below 1 × 10^−13^ scc/s (Fig. 4). These findings help constrain the potential for undesired sample loss, contamination, and chemical alteration of samples collected and sealed on Mars, as related to the leak rate. Greater leak rates are shown to correspond with higher ambient temperatures, but there does not appear to be any systematic relationship between He leak rates and tube sealing temperatures (Fig. 4). Nonetheless, the lowest He leak rates measured here are linked to sample tubes sealed at relatively high temperatures (>20°C), such as sample tube SN180 and SN193 (Fig. 4), similar to the sealing temperature of 40°C on the Perseverance rover. There is no apparent relationship between He leak rates and tube wall thicknesses or diametric tooth interferences (Supplementary Information, Figs. S1 and S2); however, the highest leak rates measured at room temperature are from sample tube SN282 (Tables 2 and 3), which is associated with the lowest tube wall thickness and diametric tooth interference. Although these engineering metrics do not appear to drastically affect the seal leak rates, it is difficult to assess their relative effects based on these data. Other factors that could contribute to seal leak rates such as obstruction from dust particles, abrasion during coring, or misalignment during sealing have also been addressed (Redmond *et al.,* 2020). In particular, dust accumulation at the top of the sample tube or around the seal area could adversely impact the leak rates by obstructing the contact between the seal cup and tube wall (Chu *et al.,* 2017). Other parameters related to sample return include static and shock loading related to the impact of the Earth Entry System at the ground recovery site; drop tower tests are currently being planned for evaluation of the tube leak rates before and after impact.

**Table 3.** Results of ^4^He Measurements for Sample Tube SN282, Integrated Over Various Accumulation Times and Listed in Descending Order of Average Run Temperatures

Average temperature (°C)	^4^He (ncc)	^4^He ncc (corrected)	Accumulation time (h)	Leak rate (scc/s)
42	11.84	11.78	23.2	1.4E-13
29.5	18.77	18.63	71.5	7.3E-14
23	11.78	11.63	69.5	4.7E-14
23	6.97	6.87	48.0	4.0E-14
23	6.45	6.38	35.2	5.1E-14
23	8.00	7.90	46.1	4.8E-14
1	1.50	1.46	19.5	2.1E-14
-1	0.72	0.67	20.5	9.6E-15
-9	0.36	0.31	22.5	4.4E-15
-17	0.30	0.27	12.5	6.9E-15
-51	0.18	0.11	29.0	1.7E-15

The measured ^4^He amounts were corrected by subtraction of the blank values.

Given the leak rates reported here, we discuss below the worst-case scenarios for the preservation of any atmospheric samples collected by Perseverance. In this regard, it is noteworthy that any potential leakage of a sample tube on Mars is expected to be negligible given the minor pressure fluctuations at the planet's surface and the resulting small pressure differential between the tubes and surrounding atmosphere. As such, the main periods of concern for sample loss, contamination, and chemical alteration are during the transit in space from Mars to Earth (when there will be vacuum outside the tubes) and following the return to Earth (when there will be ∼1 bar of terrestrial atmospheric pressure outside the tubes).

### 4.1. Transit in space

The pressure difference between the sealed sample tubes (equivalent to the average pressure on the surface of Mars of ∼5 to 7 mbar) and the vacuum of space (∼0 bar) could result in the movement of gas molecules over time from the inside of the tube to the outside. Assuming free molecular flow, Swindle *et al.* (2022) provided the following equation for estimating the amount of gas leaked from an atmospheric sample sealed on Mars for a given leak rate:

(1) $$n\left(t\right)=n_{0}exp\left(-Kt\right)$$

where *n*_0_ represents the initial gas abundance in the sample tube, *n*(*t*) is the remaining gas abundance after time *t,* and *K* is a constant determined from the known He leak rate and the sample tube volume (12.5 cc). For a He leak rate of 1 × 10^−8^ scc/s, which is the maximum allowed by the requirements for the Mars 2020 mission:

$$K=\frac{\left(1\times 10^{-8}scc∕s\right)}{\left(12.5cc\right)}K=8.0\times 10^{-10}s^{-1}$$

Thus, over the course of two years in space (*i.e.,* the maximum time period for transit from Mars to Earth), the leakage of He from the inside of the tube to high vacuum on the outside would result in

n2yr=n0exp−8.0×10−10s−1×6.3×107s=n0 exp−0.0504=0.95n0

The above calculation implies that of the original amount of He gas, approximately 95% would be retained inside the sample tube. If the amount of time that the sample tube remains in space is extended to 20 years (*e.g.,* because return of the Mars-orbiting sample container was delayed), then roughly 40% of the initial sample would be lost.

If one assumes a He leak rate of 1 × 10^−10^ scc/s (the limit verified during sample tube seal development), the amount of sample lost under these conditions decreases significantly, and after 20 years in space approximately 99.5% of the original sample would still remain. In comparison, the highest He leak rate (*i.e.,* worst-case) observed in this study (∼1 × 10^−13^ scc/s) is 3 orders of magnitude lower and indicates that >99.99% of the initial gas in a sample tube would be retained even after 50 years in space.

### 4.2. On return to Earth

Once the sample tubes have been returned to Earth, the pressure difference between the inside of the sample tube and the ambient pressure of Earth's atmosphere (∼1 bar) will generally lead to gas leakage from outside the sample tube to the inside, warranting concerns about potential terrestrial contamination. In the case of molecular nitrogen (N_2_), the partial pressure gradient will also favor the movement of N_2_ molecules from the outside to the inside of the sample tube, thereby potentially contributing to contamination of ^14^N and ^15^N between the terrestrial atmosphere (78% N_2_) and a sealed sample of martian gas (2.5% N_2_) (Tosca *et al.,* 2022). The δ^15^N composition of N_2_ in the martian atmosphere was measured by NASA's Curiosity rover (δ^15^N_Mars_ = 572 ± 82‰; Wong *et al.,* 2013) as being isotopically distinct from terrestrial air (by definition, δ^15^N_air_ = 0‰).


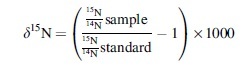


A leak in the seal of a sample tube containing martian atmosphere would be expected to result in the preferential loss (when in space) or gain (when on Earth) of the lighter isotope of nitrogen, thereby potentially compromising the δ^15^N value that would be measured and the subsequent interpretation of source reservoirs and atmospheric processes.

For an individual sample tube filled with martian atmosphere, Swindle *et al.* (2022) estimated that 2.35 × 10^−3^ cc (1.05 × 10^−7^ mol) of N_2_ will be contained within a sample tube with a volume of 12.5 cc. Therefore, to change the isotopic composition of N_2_ in a sample of martian atmosphere by <1‰, no more than 4.7 × 10^−6^ cc (2.1 × 10^−10^ mol) of N_2_ can leak into the tube. Assuming a sample tube with a leak rate of 1 × 10^−8^ scc/s for air, the amount of time required to change the δ^15^N composition of martian gas by 1‰ from the addition of terrestrial atmosphere is approximately 10 min, and this length of time increases to about 16 h for a leak rate of 1 × 10^−10^ scc/s. Using the highest He leak rate measured here (∼1 × 10^−13^ scc/s, which is the worst case given that the expected leak rate for air would be lower than for He gas), the estimated amount of time needed to impart a 1‰ change to the δ^15^N of martian gas through a leak in the seal would be roughly 2 years.

Other atmospheric gases will behave differently and are expected to have lower leak rates in comparison with the measurements made using He in this study. For example, assuming free molecular flow and a similar leak path geometry, it was determined that water vapor (H_2_O) will have a lower leak rate in the sample tubes due to differences in physical properties of the gases (Redmond *et al.,* 2015). Conversion factors are known for other gases, including nitrogen, argon (Ar), and air, for both viscous and molecular flow regimes (see Oravec *et al.,* 2017). Water vapor is typically very low in abundance and relatively variable on Mars, generally representing less than 0.03% of the atmosphere, whereas carbon dioxide (CO_2_) comprises 95% and is the dominant atmospheric gas on Mars. In comparison, N_2_ and Ar represent about 2.5% and 2% of the martian atmosphere, respectively, with other gases present in lower abundances (Trainer *et al.,* 2019). Thus, the gas contained within an atmospheric sample tube collected on Mars will be mixed and mostly CO_2_. Most gases of interest will leak in the direction of the overall pressure gradient, and the main task upon successful sample return will be minimizing the amount of time that the unopened sample tube seals are exposed to terrestrial atmosphere.

## 5. Conclusions

Our results extend previous findings that the Mars 2020 sample tube seals are likely to be characterized by very low He leak rates, roughly 3 to 6 orders of magnitude below the previously quoted detection limit of 10^−10^ scc/s. Given this, leakage of the tube seals can be ignored for most practical applications. Returning samples of rocks, regolith, and atmospheric gases collected *in situ* on Mars will represent a historic milestone for the fields of planetary science and astrobiology. Maintaining these samples in a condition no worse than when they are collected on Mars is, therefore, of paramount importance, beginning with the collection of hermetically sealed samples on board the Perseverance rover and lasting throughout their planned transit in space until they are delivered to Earth as part of the MSR campaign. Further studies are needed to fully assess the underlying physics in these relevant environments, as well as for different leak paths and flow regimes. However, the results presented here can be used to inform potential sample handling and curation requirements, including thermal management considerations and the time frame for sample retrieval and containment after their return to Earth. This empirical study of He leak rates for flight-like sample tubes serves to build confidence in the reliability of the hermetic seals and reaffirm their suitability for preserving the integrity of the samples collected by Perseverance on the surface of Mars.

## Supplementary Material

Supplemental data

Supplemental data

## Abbreviations Used

JPL: Jet Propulsion Laboratory
MSR: Mars Sample Return
ncc: nano-cubic centimeters
scc/s: standard cubic centimeters per second
TiN: titanium nitride
UHP: Ultra High Purity

## Notes

Associate Editor: John Rummel
