# Supplementary material for: Helium Leak Rate Measurements of Flight-like Mars 2020 Sample Tubes
Source: Astrobiology. 2024 Jan 12;24(1):36–43. doi: 10.1089/ast.2023.0002 (PMC10795500; doi:10.1089/ast.2023.0002)
Supplement: Supplemental data [file Suppl_FigS2.pdf]

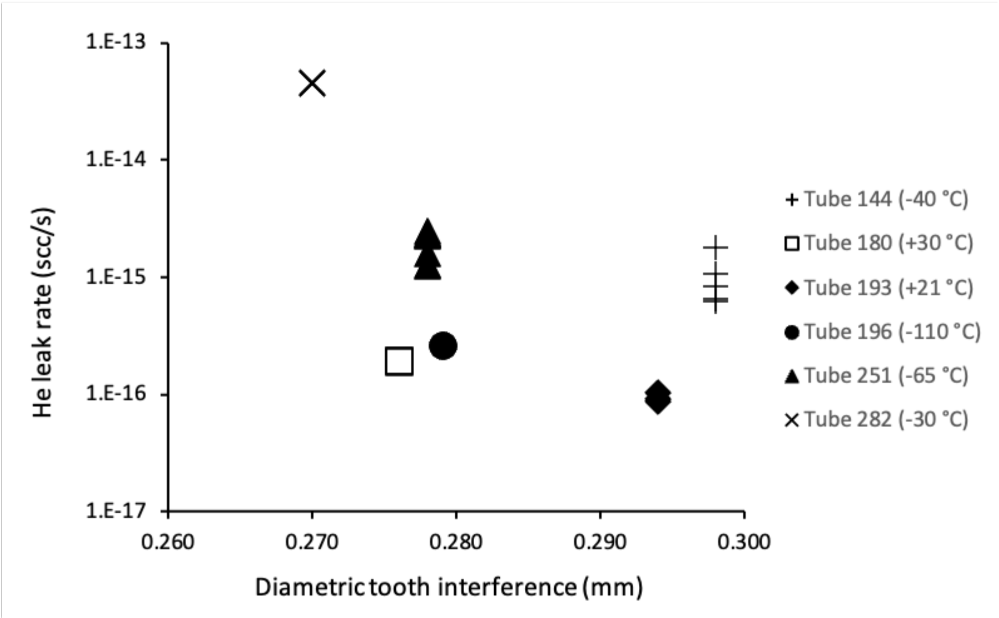

Figure S2. Results of measurements of He leak rates for six flight-like sample tubes with varying diametric tooth interferences. Vertical axis is plotted on a logarithmic scale; some data points overlap and are indistinguishable on this graph.
